# Risk Factors of Venous Thromboembolism in Inflammatory Bowel Disease: A Systematic Review and Meta-Analysis

**DOI:** 10.3389/fmed.2021.693927

**Published:** 2021-06-28

**Authors:** Hua Zhang, Xuehong Wang

**Affiliations:** ^1^Department of Nursing, Xiangya Second Hospital of Central South University, Changsha, China; ^2^Department of Gastroenterology, Xiangya Second Hospital of Central South University, Changsha, China

**Keywords:** inflammatory bowel disease, risk factors, venous thromboembolism, systematic review, meta-analysis

## Abstract

**Background:** Patients suffering from chronic inflammatory disorders, such as inflammatory bowel disorder, are at higher risk of developing thromboembolism. The chronic inflammatory nature of inflammatory bowel disease has been identified as a predominant reason for a state of Virchow's triad (i.e., endothelial dysfunction, stasis, and general hypercoagulability), eventually leading to the onset of venous thromboembolism. Recent studies show that certain factors, such as demographics, medication history, and history of surgical intervention may increase thromboembolism risk in patients with inflammatory bowel disease. However, to date, no study has attempted to evaluate the effect of different risk factors associated with the development of venous thromboembolism in inflammatory bowel disease patients.

**Objective:** To evaluate the risk factors that can influence the incidence of venous thromboembolism in patients with inflammatory bowel disease.

**Methods:** Academic literature was systematically searched based on the PRISMA guidelines across five databases: Web of Science, EMBASE, CENTRAL, Scopus, and MEDLINE. A random-effect meta-analysis was conducted to evaluate the hazard ratio for the risk factors (i.e., aging, gender, steroid therapy, surgery, and ulcerative colitis) that can influence the incidence of venous thromboembolism in patients with inflammatory bowel disease.

**Results:** From a total of 963 studies, 18 eligible studies with 1,062,985 (44.59 ± 10.18 years) patients suffering from inflammatory bowel disease were included in the review. A meta-analysis revealed a higher risk of aging (Hazard's ratio: 2.19), steroids (1.87), surgery (1.48), and ulcerative colitis (2.06) on venous thromboembolism in patients with inflammatory bowel disease. We also found that the female gender (0.92) did not increase the incidence of venous thromboembolism in inflammatory bowel disease patients.

**Conclusion:** The study provides preliminary evidence regarding high risks associated with ulcerative colitis, steroid consumption, and aging for the development of venous thromboembolism in patients with inflammatory bowel disease. The findings from this study may contribute to developing awareness among clinicians, better risk stratification and prevention of venous thromboembolic complications in patients with inflammatory bowel disease.

## Introduction

Inflammatory bowel disease is one of the most frequent types of chronic inflammatory disorders in the world (1, 2), with 6.8 million cases worldwide (3), and 84.3 per 100,000 people affected (4, 5). According to the Center for disease control and prevention, inflammatory bowel disease is primarily characterized as a chronic inflammatory disorder of the gastrointestinal system, affecting usually the small and the large intestine (6).

Inflammatory bowel disease is a term that describes two conditions, Crohn's disease and ulcerative colitis (7). Depending on the type of inflammatory bowel disease, the inflammatory changes can either inflict substantial damage to the mucosal lining of the colon i.e., ulcerative colitis, or any part of the gastrointestinal tract i.e., Crohn's disease (8, 9). Besides, in situations where distinction between ulcerative colitis and Crohn's disease difficult (i.e., around 10–15% of cases) the condition is termed as indeterminate colitis (10). Typically, the denudation of the mucosal layer due to the downregulation of proteins released by goblet cells can weaken the epithelial layer leading to an increased penetration of foreign microbiota (8, 11) and subsequent local inflammatory reaction (12). This, in turn, promotes the release of pro-inflammatory signals, increased concentration of leukocytes and chemokines at the site of lesion, and a pronounced T-cell immune response (13, 14). In addition to affecting the gastrointestinal site, this intestinal disorder also causes an inadvertent activation of the systemic inflammatory (15, 16). Irving et al. (17) described that the release of inflammatory markers (i.e., cytokines, tumor necrosis factor-α, interleukin-1β) at the affected gastrointestinal site into the circulatory system could impair the functioning of the endothelial system, promote hypercoagulability, and induce stasis. In addition to the inflammatory markers, increased levels of fibrinogen, D-dimer, von Willebrand-factor, anti-endothelial antibodies could also lead to a cascading coagulation effect and promote thromboembolic events (17, 18). Numerous reports show that the risk of a venous thromboembolic event in patients with inflammatory bowel disease may be further aggravated by additional acquired risk factors, such as steroid therapy, smoking, aging, obesity, immobilization, and central venous catheters (19). Studies have suggested that these risk factors can inadvertently promote a hyper-coagulative phase that might increase the incidence of venous thromboembolism (20, 21), eventually worsening the morbidity- and mortality-related outcomes for patients with inflammatory bowel disease (22, 23).

To date, several retrospective cohort studies (24–29) have attempted to evaluate the effect of acquired risk factors on the incidence of venous thromboembolic events in inflammatory bowel disease patients with controversial results. For instance, while some studies had reported a significantly increased risk in aging inflammatory bowel disease patients (24, 27, 30), other studies did not report this outcome (28, 31, 32). Similarly, there is still a lack of consensus regarding the risks of venous thromboembolism associated with steroid therapy, surgery, obesity in patients with inflammatory bowel disease (24, 28, 29, 33–35). To the best of our knowledge, there are no systematic reviews or meta-analyses that summarized the existing data, evaluating risk factors that can influence the incidence of venous thromboembolism in patients with inflammatory bowel disease.

In the present systematic review and meta-analysis, we attempt to summarize current state of evidence regarding the overall risk factors associated with an increased incidence of venous thromboembolism in patients with inflammatory bowel disease. The findings from the present study may contribute to increased clinical awareness of different risk factors that might predispose patients with inflammatory bowel disease toward venous thromboembolism.

## Methods

Meta-analysis was conducted in adherence to PRISMA (Preferred Reporting Items for Systematic Reviews and Meta-Analyses) guidelines (36).

### Data Search Strategy

The literature search was carried out in five scientific databases (Web of Science, MEDLINE, CENTRAL, EMBASE, and Scopus) from inception till 15th January 2021. The search was performed across a combination of MeSH keywords including “Inflammatory bowel disease,” “risks,” “steroids,” “surgery,” “obesity,” “ulcerative colitis,” “Crohn's disease,” “venous thromboembolism,” and “thromboembolism.” A detailed search strategy for the EMBASE database has been provided in Supplementary Table 1. The bibliography section of the included studies was manually searched to further identify additional studies. The inclusion criteria were:

a) Studies that evaluate the risks of age, female gender, steroids, surgery, obesity, and ulcerative colitis in patients with inflammatory bowel disease.
b) Studies of human participants.
c) Case-control studies, prospective cohort trials, or retrospective cohort trials.
d) Studies published in peer-reviewed scientific journals.
e) English language studies.

The screening of the studies was independently performed by two reviewers.

### Quality Assessment

Risk of bias in the selected manuscripts was assessed by Cochrane's risk of bias assessment tool for non-randomized controlled trials (37). This tool evaluates the outcomes for selective reporting, confounding bias, measurement of outcomes, and incomplete data availability. The appraisal of methodological quality was performed independently by two reviewers.

### Data Analysis

A within-group meta-analysis was performed using CMA, Comprehensive Meta-analysis version 2.0 (38), based on the random-effects model (39). We calculated the hazard ratio to determine the risks of age, female gender, steroids, surgery, obesity, and ulcerative colitis for patients with inflammatory bowel disease. Heterogeneity among the studies was assessed by computing I^2^ statistics. I^2^ statistics between 0 and 25% was considered indicative of negligible heterogeneity, 25–75% of moderate heterogeneity, and ≥75% of substantial heterogeneity (40). Publication bias was evaluated by Duval and Tweedy's trim and fill procedure (41) that identifies any unbiased effect by imputing studies from either side of the plotted graph. The level of significance for this study was aloocated at 5%.

## Results

**Figure 1:**
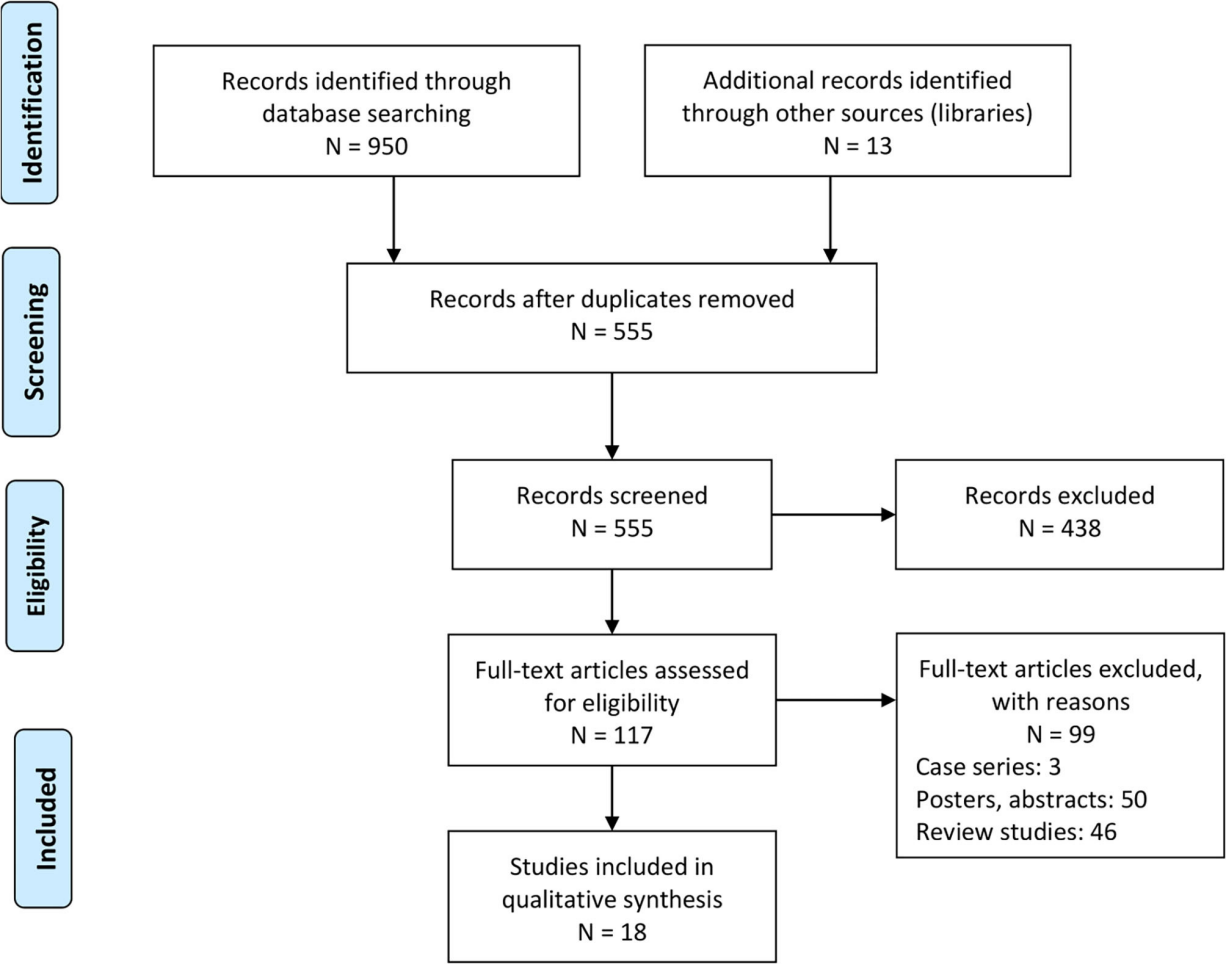
PRISMA flowchart.

A search across five academic databases provided 950 studies. We identified an additional 13 during the screening of the reference sections of the included studies. Eighteen studies met inclusion criteria; 17 of the included studies were retrospective cohort studies (22, 24–32, 34, 35, 42–47), one of the included studies was a case-control study (48) (Figure 1). The data was extracted in a tabular format and has been summarized in Table 1.

**Table 1:** Demonstrates the details of the included studies.

**Study**	**Country**	**Type of study**	**IBD sample descriptive**	**Total VTE events**	**Age (M ± S.D years)**
Schlick et al. (29)	USA	Retrospective cohort study	18990 (9577F, 9413M)	199	42.7 ± 16.7
Liu et al. (48)	Canada	Case control study	8459 (3151F, 5308M)	46	43.1
Faye et al. (26)	USA	Retrospective cohort study	872122 (498604F, 373518M)	1,160	–
Ohta et al. (28)	Japan	Retrospective cohort study	72 (24F, 49M)	43	36.6 ± 15.3
McCurdy et al. (27)	Canada	Retrospective cohort study	2161 (1154F, 1007M)	6	47.3
Andrade et al. (22)	Brazil	Retrospective cohort study	1093 (627F, 467M)	37	–
Beal et al. (45)	USA	Retrospective cohort study	77823 (40653F, 37170M)	60	62
Benlice et al. (44)	USA	Retrospective cohort study	24182 (11968F, 12214M)	614	43
Ando et al. (24)	Japan	Retrospective cohort study	340 (106F, 234M)	24	39.4 ± 15.4
Chu et al. (25)	UK	Retrospective cohort study	23046 (12228F, 10818M)	430	54.1
Alatri et al. (31)	Switzerland	Retrospective cohort study	2284 (1133F, 1151M)	90	28
Pellino et al. (43)	Italy	Retrospective cohort study	146 (146F)	1	19–38
Vegh et al. (34)	Hungary	Retrospective cohort study	1708 (829F, 879M)	22	22–50
Nelson et al. (46)	USA	Retrospective cohort study	16120 (8780F, 7340M)	360	61.4 ± 15.7
Ananthakrishnan et al. (32)	USA	Retrospective cohort study	2788 (163F, 2625M)	760	–
Allaix et al. (47)	USA	Retrospective cohort study	1069 (500F, 569M)	37	41.5 ± 15.8
Nguyen et al. (42)	Canada	Retrospective cohort study	150 (72F, 78M)	49	36
Wallaert et al. (35)	USA	Retrospective cohort study	10431 (4999F, 5432M)	242	–

*M, Mean; S.D, Standard deviation; F, Female; M, Male; IBD, Inflammatory bowel disease; CD, Chron's disease; UC, Ulcerative colitis*.

### Participant Information

Data from a total of 1,062,985 (594714F, 468272M) patients were evaluated in the 18 included studies. In the entire cohort, 4,789 events of venous thromboembolism were reported.

The average age of the participants was as 44.59 ± 10.18 years. In three studies sample age was reported as a range (33, 34, 43), and four studies did not report the descriptive information concerning the age of their sample (22, 26, 32, 35).

### Assessment for Quality in Non-randomized Controlled Trials

**Figure 2:**
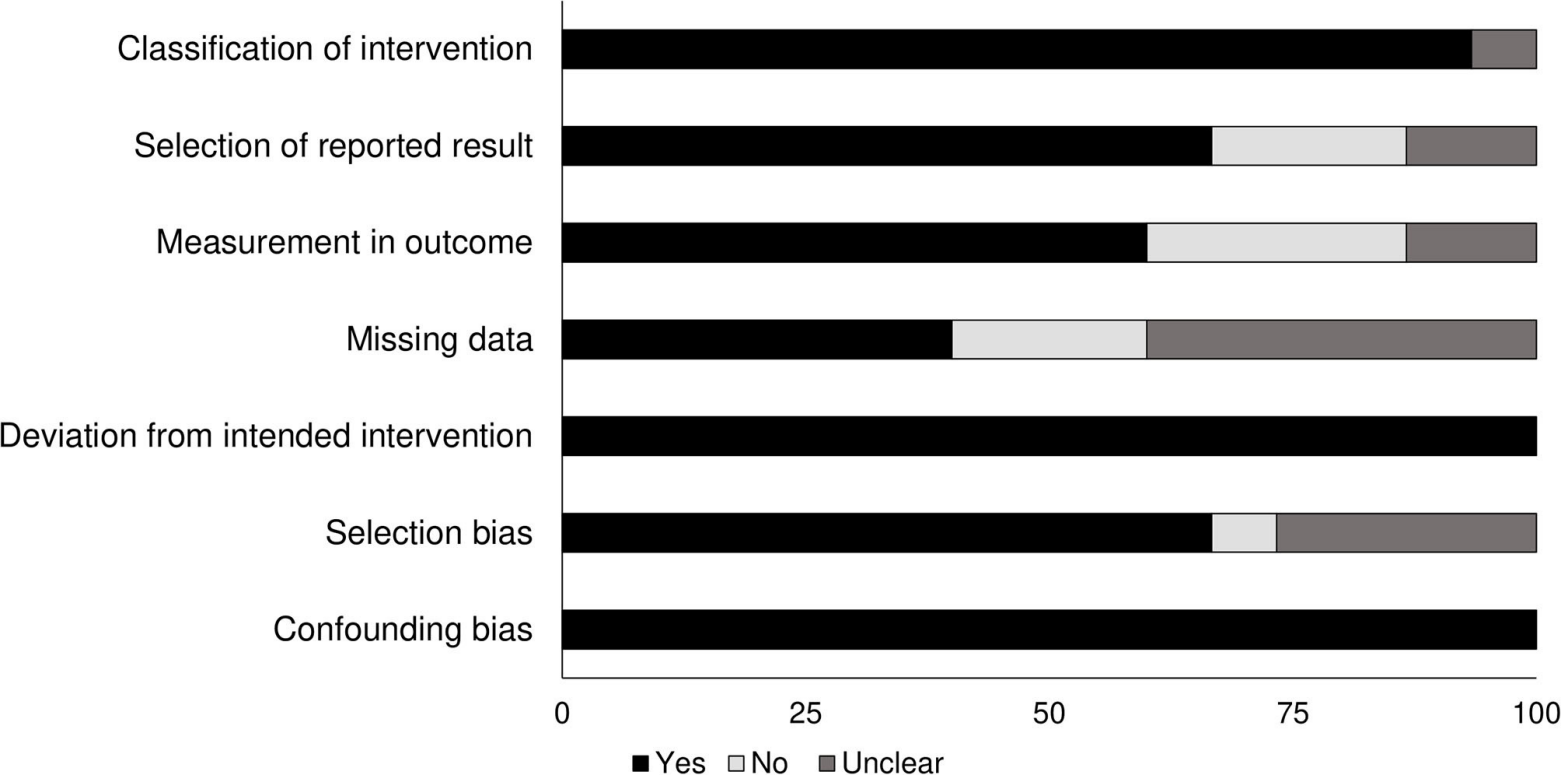
Demonstrates the risk of bias according to the Cochrane risk of bias assessment for the randomized controlled trials.

The risk of bias in the methodology of the non-randomized controlled trials was evaluated with the ROBINS I appraisal tool (summarized in Table 2). The overall risk of bias was low in all the included studies. The main categories with the detected bias included missing data, selection of reported results, measurement in the outcome, and selection bias. The overall risk of bias also is shown in Figure 2.

**Table 2:** Demonstrates the risk of bias according to Cochrane's risk of bias assessment tool for randomized controlled trials.

**Study**	**Confounding bias**	**Selection bias**	**Deviation from intended intervention**	**Missing data**	**Measurement in outcome**	**Selection of reported result**	**Classification of intervention**
Schlick et al. (29)	+	+	+	+	+	+	+
Liu et al. (48)	+	+	+	+	+	+	+
Faye et al. (26)	+	?	+	–	?	–	+
Ohta et al. (28)	+	+	+	+	+	+	?
McCurdy et al. (27)	+	+	+	+	+	+	+
Andrade et al. (22)	+	+	+	–	?	?	+
Beal et al. (45)	+	+	+	+	+	+	+
Benlice et al. (44)	+	+	+	?	?	?	+
Ando et al. (24)	+	+	+	?	+	+	+
Chu et al. (25)	+	+	+	?	+	+	+
Alatri et al. (31)	+	–	+	–	–	–	+
Pellino et al. (43)	+	?	+	?	+	+	+
Vegh et al. (34)	+	?	+	?	+	+	+
Nelson et al. (46)	+	?	+	?	–	–	+
Ananthakrishnan et al. (32)	+	?	+	?	+	+	+
Allaix et al. (47)	+	?	+	?	+	+	+
Nguyen et al. (42)	+	?	+	?	+	+	+
Wallaert et al. (35)	+	?	+	?	+	+	+

### Publication Bias

**Figure 3:**
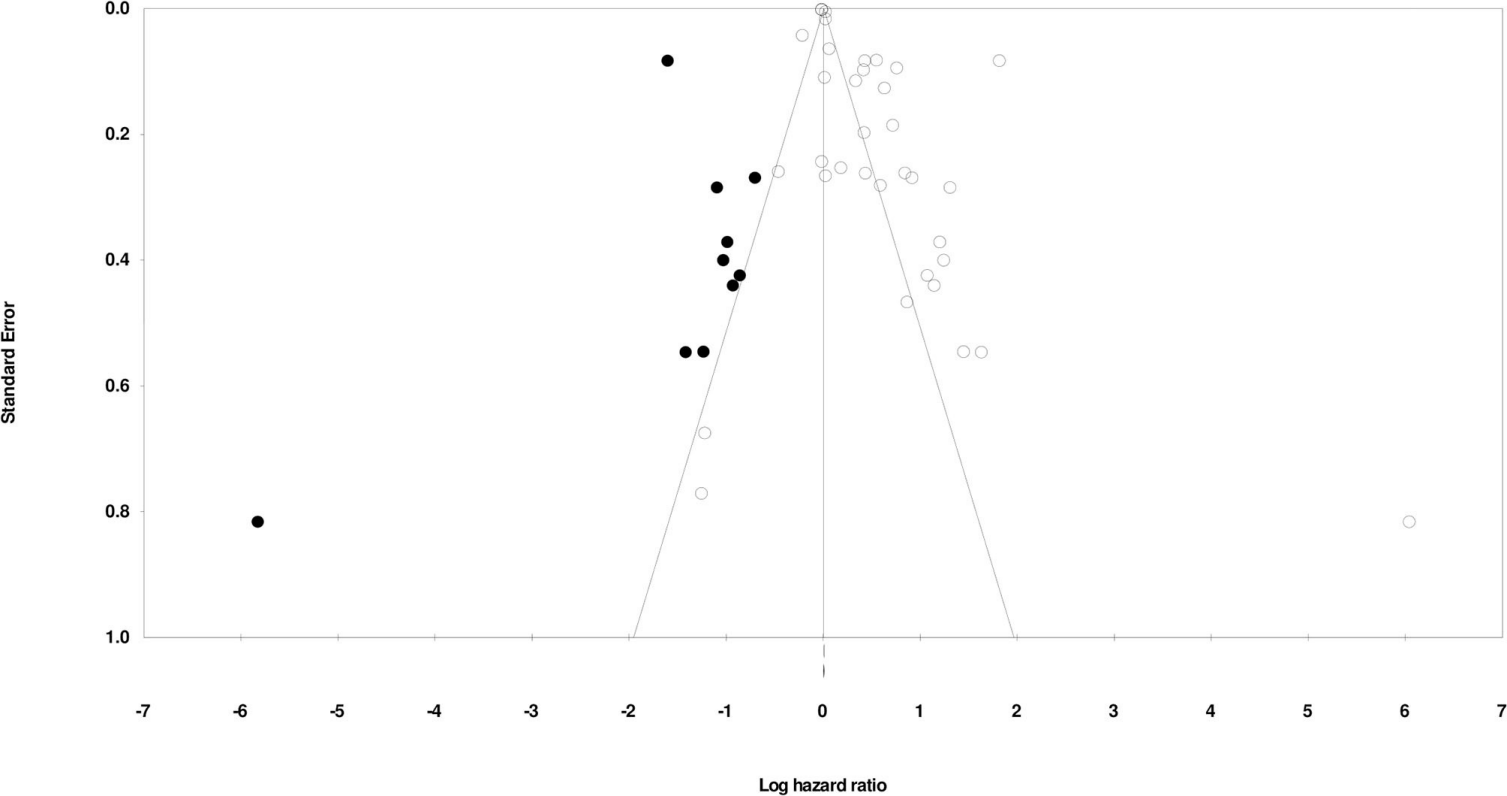
Demonstrates the publication bias by Duval & Tweedy's trim and fill method.

We used Duval and Tweedy's trim and fill method to determine missing studies on either side of the mean effect of the funnel plot using to the random effect model. There were 10 studies missing on the left side of the mean effect. We evaluated point estimates and the 95% confidence intervals on the basis of random effect model for all the combined studies were calculated as 1.37 (1.30–1.45), using the trim an fill the imputed point estimate is 1.21 (1.41–1.28). The publication bias is reported in Figure 3.

### Meta-Analysis Report

#### Age

**Figure 4:**
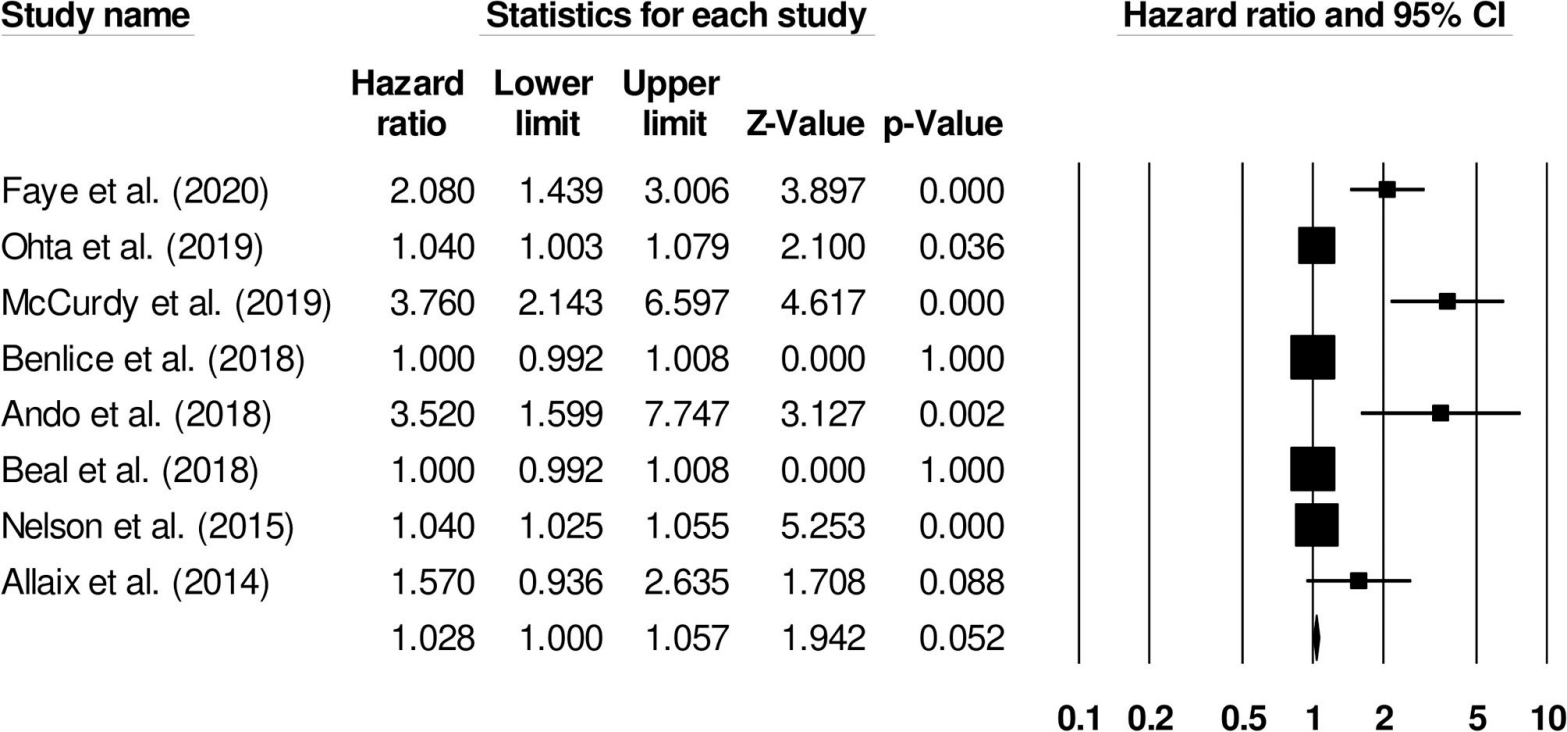
Forest plot for studies evaluating age as a risk factor of venous thromboembolism in patients with inflammatory bowel disease. The hazard ratios are presented as black boxes; 95% confidence intervals are presented as whiskers. A small hazard ratio represents lower risks of venous thromboembolism with age, and a higher hazard ratio represents higher risks of venous thromboembolism with age in patients with inflammatory bowel disease.

The association of age with the risk of venous thromboembolism in inflammatory bowel disease patients was reported by eight studies. An increased risk of venous thromboembolism was observed in older patients (Figure 4) (1.02, 95% C.I: 1.0–1.05, *p* = 0.052), with substantial heterogeneity (I^2^: 85.4%).

#### Female Gender

**Figure 5:**
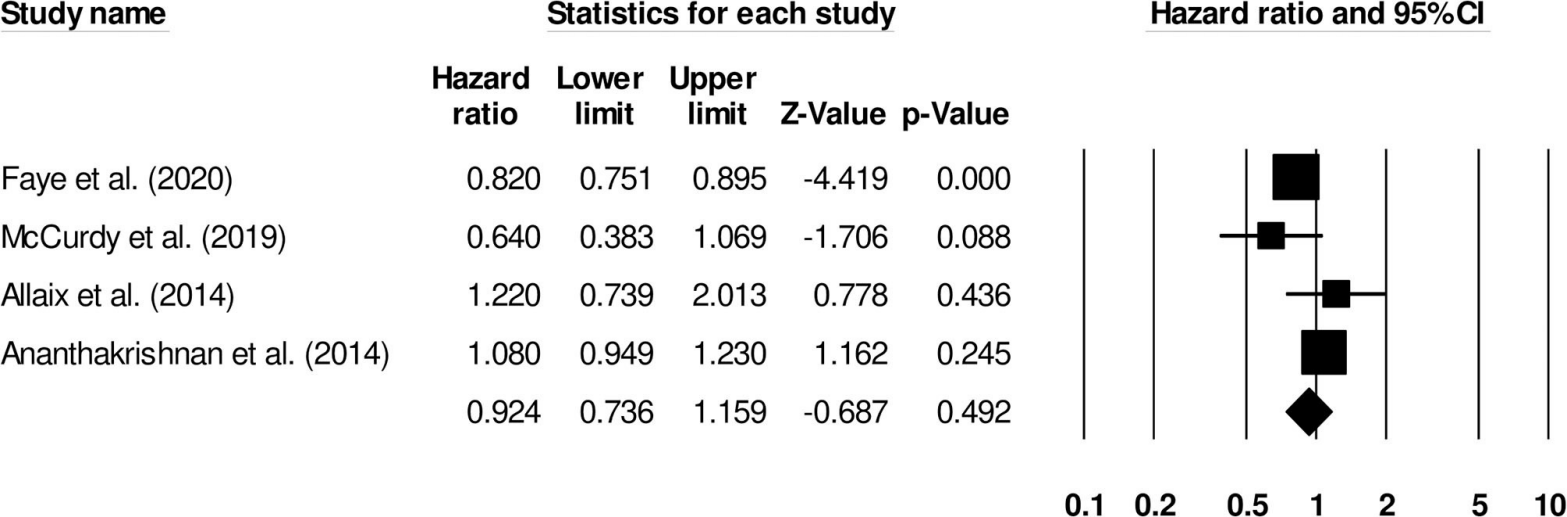
Forest plot for studies evaluating female gender as a risk factor of venous thromboembolism in patients with inflammatory bowel disease. The hazard ratios are presented as black boxes whereas 95% confidence intervals are presented as whiskers. A small hazard ratio represents lower risks of venous thromboembolism associated with female gender, and a higher hazard ratio represents higher risks of venous thromboembolism associated with female gender in patients with inflammatory bowel disease.

The correlation between the female gender and the risk of venous thromboembolism in inflammatory bowel disease patients was reported by four studies. There was no significant effect of female gender on the risk of venous thromboembolism (Figure 5) (0.92, 95% C.I: 0.73–1.15, *p* = 0.49), with negligible heterogeneity (I^2^: 4.8%).

#### Steroids

**Figure 6:**
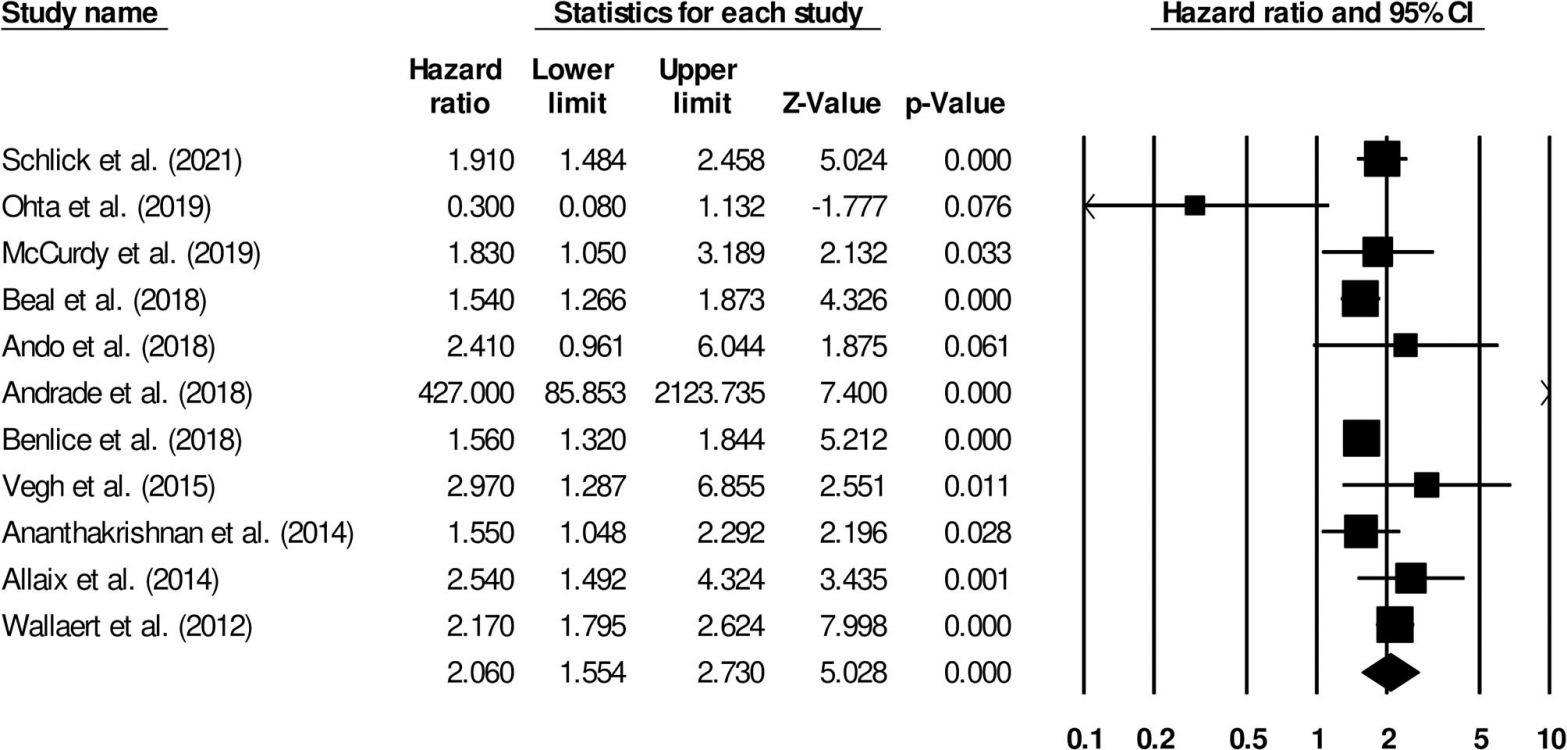
Forest plot for studies evaluating the effect of steroids on the risk of venous thromboembolism in patients with inflammatory bowel disease. The hazard ratios are presented as black boxes; 95% confidence intervals are presented as whiskers. A small hazard ratio represents lower risks of venous thromboembolism in patients with inflammatory bowel disease, administered steroid drugs; a higher hazard ratio represents higher risks of venous thromboembolism in patients with inflammatory bowel disease, administered steroid drugs.

The association between the risk of venous thromboembolism and steroids in patients with inflammatory bowel disease was reported by 11 studies. There was a significantly higher risk of venous thromboembolism in patients that received steroids (Figure 6) (2.06, 95% C.I: 1.55–2.73, *p* < 0.01), with moderate heterogeneity (I^2^: 76.8%).

#### Surgery

**Figure 7:**
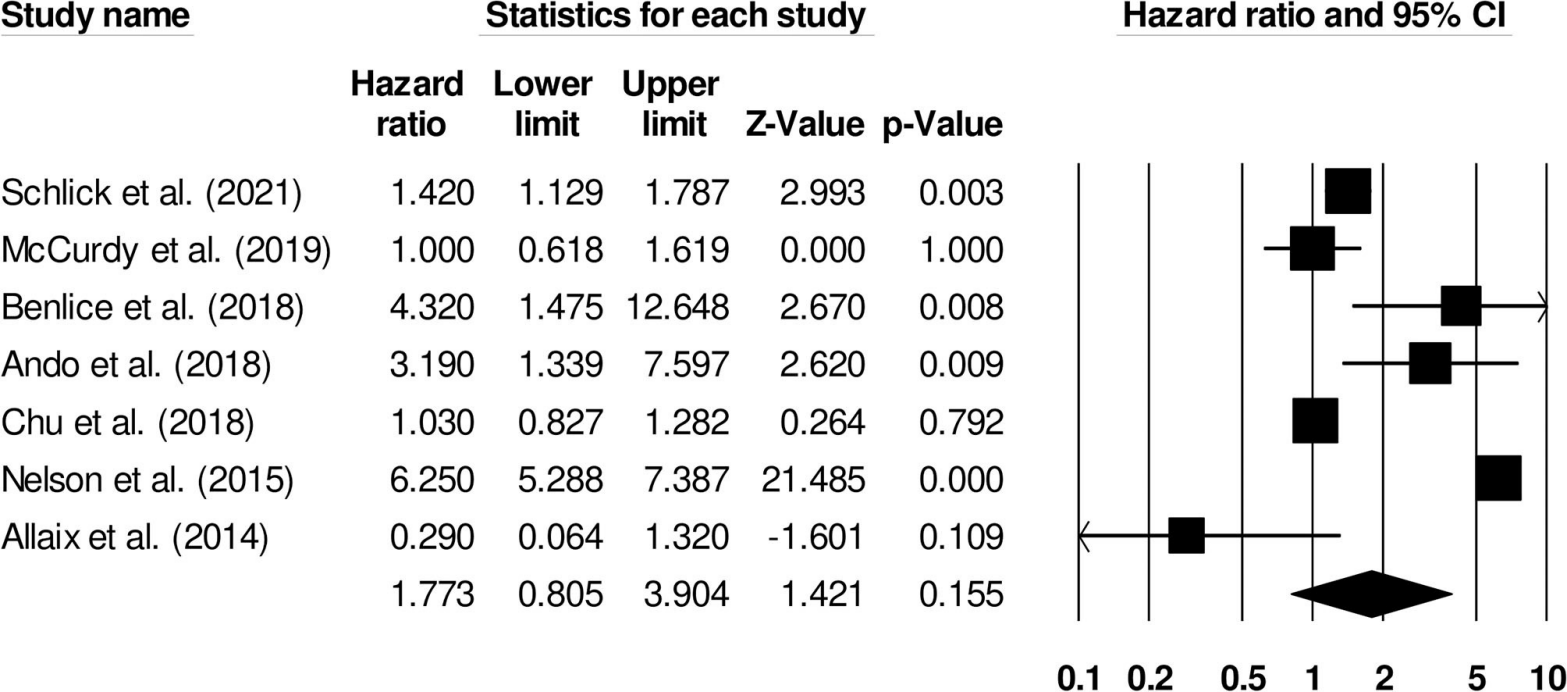
Demonstrates the forest plot for studies evaluating the risk of surgery on venous thromboembolism in patients with inflammatory bowel disease. The hazard ratios are presented as black boxes whereas 95% confidence intervals are presented as whiskers. A small hazard ratio represents lower risks of surgery on venous thromboembolism in patients with inflammatory bowel disease, a higher hazard ratio represents higher risks of surgery on venous thromboembolism in patients with inflammatory bowel disease.

The increased risk of venous thromboembolism in surgical patients with inflammatory bowel disease was reported by seven studies. We observed an increased risk of venous thromboembolism in this group of patients (Figure 7) (1.77, 95% C.I: 0.80–3.90, *p* = 0.15), with no heterogeneity (I^2^: 0%).

#### Ulcerative Colitis

**Figure 8:**
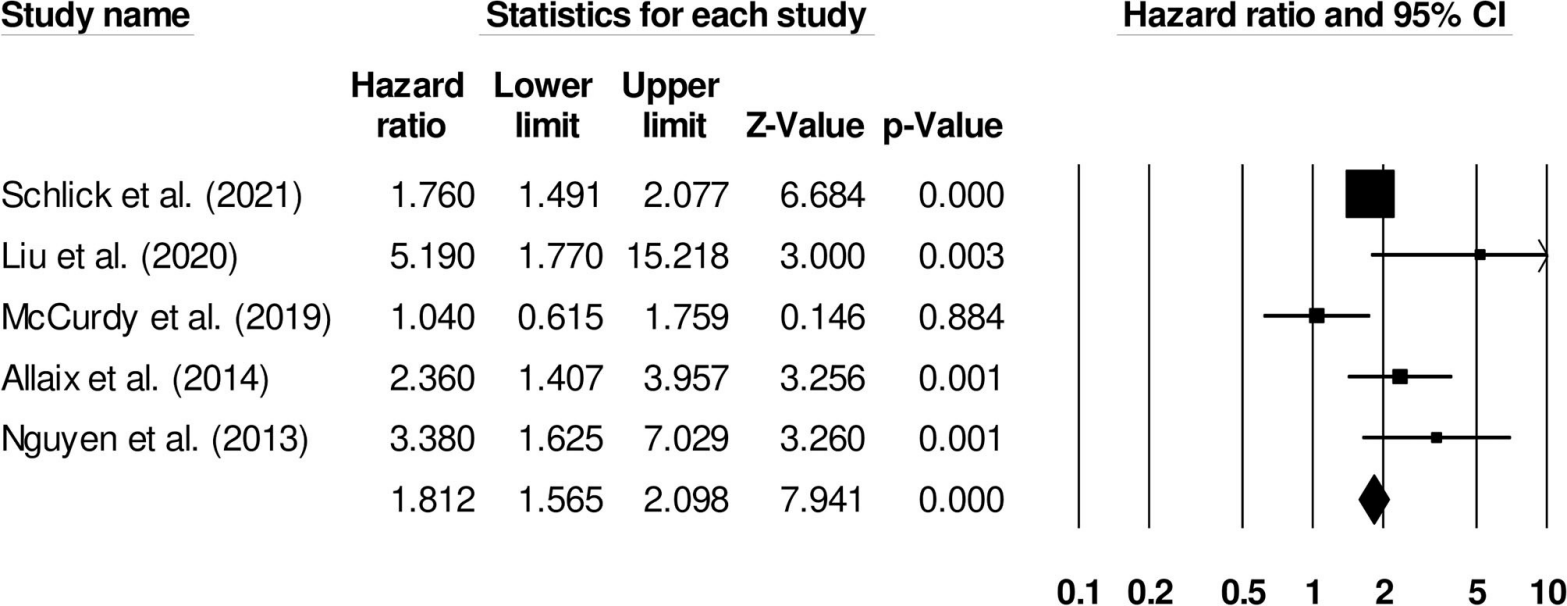
Forest plot for studies evaluating the effect of ulcerative colitis on the risk of venous thromboembolism in patients with inflammatory bowel disease. The hazard ratios are presented as black boxes whereas 95% confidence intervals are presented as whiskers. A small hazard ratio represents lower risks of venous thromboembolism in ulcerative colitis patients with inflammatory bowel disease, a higher hazard ratio represents higher risks of venous thromboembolism in ulcerative colitis patients with inflammatory bowel disease.

The risk of venous thromboembolism in inflammatory bowel disease patients with ulcerative colitis was reported by five studies. We observed an increased risk of venous thromboembolism in ulcerative colitis patients (Figure 8) (1.81, 95% C.I: 1.56–2.09, *p* < 0.001), with moderate heterogeneity (I^2^: 66.3%).

## Discussion

To our knowledge, this study is the first systematic review and meta-analysis that provides comprehensive evidence regarding the risk factors associated with the increased incidence of venous thromboembolism in patients with inflammatory bowel disease. We observed increased risk of venous thromboembolism associated with aging, steroid therapy, surgery, obesity, and ulcerative colitis in patients with inflammatory bowel disease. We also reported that in this patients the female gender did not affect the incidence of venous thromboembolism.

The management of inflammatory bowel disease is challenging because of its atypical pathophysiological mechanism, co-existing morbidities, and manifestations (49–51). Higher predisposition to venous thromboembolic events is one of the common co-morbidities of patients affected with inflammatory bowel disease (15, 20, 21) and is associated with poor prognostic outcome in terms of short-, long-term morbidity and mortality (22, 52). Studies indicate that the onset of thromboembolic events in patients with inflammatory bowel disease is a result of multifactorial mechanisms that cause the development of local and/or systemic inflammation due to endothelial dysfunction, vascular infiltration of inflammatory monocytes, and upregulation of thrombogenic factors (53–55). These inflammatory changes lead to the development of Virchow's triad (vascular injury, hemostasis, and hypercoagulation), ultimately resulting in increased incidence of venous thromboembolism (56, 57). In the existing literature, even the therapeutic interventions (i.e., steroid therapy, abdominal surgery, central venous catheter) generally administered to manage inflammatory bowel disease have been suggested as additional predisposing factors that increase the risks of venous thromboembolism (58, 58–60). Studies have shown that these interventions can precipitate a transient hyper-coagulative phase in the vascular system which might further increase the risks of venous thromboembolism in inflammatory bowel disease patients (61, 62).

In the present systematic review, we reported several risk factors that predisposed patients toward a higher risk of developing venous thromboembolism. Our results show that treatment procedures related to inflammatory bowel disease i.e., steroid therapy, surgical interventions were among the major risk factors that increasingly predisposed patients toward venous thromboembolism. In his study of a cohort representative of the population of the United States of America, Wallaert et al. (35) showed that chronic use of steroid therapy was reported in almost 60% of inflammatory bowel disease patients with venous thromboembolism. It is possible that the modifications in the fibrinolytic/hemostatic factors due to the steroid therapy in patients with inflammatory bowel disease leads to increased incidence of venous thromboembolism (63, 64). The authors also suggested that measures, such as aggressively tapering down steroid consumption or altering the magnitude of a surgery (grade-3 instead of grade-2 ileal pouch-anal anastomosis operation) could reduce the risk of venous thromboembolism. Furthermore, in a novel study, McKie et al. (33) evaluated risks of venous thromboembolism associated with surgical interventions in adolescents with inflammatory bowel disease. The authors reported that the risks of venous thromboembolism were increased almost 2-fold in inflammatory bowel disease patients undergoing a major abdominal surgical intervention (Odds ratio, 95% C.I: 1.98, 1.54–2.55). Based on the findings of Brady et al. (61), it can be interpreted that this increased risk of venous thromboembolism post-surgery could primarily be due to a lack of chemical thromboprophylaxis. Ando et al. (24) and Schlick et al. (29) also reported the increased incidence risks of venous thromboembolism in inflammatory bowel disease patients with prior history of steroid therapy and surgery. Our meta-analysis confirms these findings and reports that the risks of venous thromboembolism were increased due to steroid therapy (1.87) and surgery (1.48) in patients with inflammatory bowel disease.

In the current review and meta-analysis, we also attempt to develop a consensus regarding the demographic risk factors, such as age and gender that can influence the outcome of venous thromboembolism in patients with inflammatory bowel disease. In a retrospective cohort study by McCurdy et al. (27), among 2,161 patients with inflammatory bowel disease, the patients over 45 years of age were more than three times predisposed to developing venous thromboembolism after discharge from the hospital (OR: 3.76, 1.80–7.89). The authors further developed a clinical risk stratification scale on which they categorized age above 45 years as a “high-risk” condition and suggested the importance of administering selective thromboprophylaxis to avoid incidences of venous thromboembolism. Similarly, Faye et al. (26) in a large cohort study of 872,122 patients reported that not only did the aging increase the risks of venous thromboembolism in patients with inflammatory bowel disease, but that it also increased the rate of venous thromboembolism-related re-admission to the hospital. The authors further reported a difference in age-related risks between patients with ulcerative colitis and Crohn's disease. Ulcerative colitis patients between the ages of 41 and 50 years had significantly higher risk of venous thromboembolism-based re-admission (Adjusted risk ratio, 95% C.I: 7.21, 1.73–30.09), as compared to patients with Crohn's disease (ARR: 1.41, 0.82–2.41). The results of our meta-analysis confirm these findings. We found higher risks of venous thromboembolism associated with aging (2.19), as well as higher risks of venous thromboembolism in patients with ulcerative colitis as compared to Crohn's disease (2.06). There was no overall effect of gender (0.92) on the venous thromboembolism incidence.

Our systematic review and meta-analysis have several limitations. First and foremost, this study is not registered in a review repository such as PROSPERO York or Joanna Briggs Institute. We understand that while the lack of prior pre-registration could have a negative effect on the validity of this current systematic review and meta-analysis (65), we assure our reviewers and our reader that several attempts were made by us to register this present review. Due to the current COVID-19 pandemic crisis, registration times at the repositories have been extended by more than 1 year. Second, we could not include studies which had evaluated the risk factors of venous thromboembolism in adolescent population groups due to paucity in the literature. This could suggest that our results are not generalizable on all population groups but only adult. Nevertheless, we recommend future cohort and case-control studies to address these limitations, while evaluating risk factors that influence the incidence of venous thromboembolism in the inflammatory bowel disease patients of all age groups, and share descriptive data in open access data repositories. Such studies would allow clinicians to develop risk stratification guidelines for reducing the morbidity- and mortality-related outcomes for venous thromboembolism in patients with inflammatory bowel disease.

In conclusion, this systematic review and meta-analysis study provide preliminary evidence of the risk factors that can affect the incidence of venous thromboembolism in patients with inflammatory bowel disease. We show that steroid therapy, aging and surgery increase risks of venous thromboembolism in patients with inflammatory bowel disease. The findings from the present study may contribute to developing best practice guidelines for managing co-existing venous thromboembolic conditions in patients with inflammatory bowel disease.

## Data Availability Statement

The original contributions generated for this study are included in the article/Supplementary Material, further inquiries can be directed to the corresponding author/s.

## Author Contributions

HZ designed the project and edited the manuscript. XW and HZ were involved in data collection and data analysis. XW prepared the manuscript. All authors have read and approved the final manuscript.

## Conflict of Interest

The authors declare that the research was conducted in the absence of any commercial or financial relationships that could be construed as a potential conflict of interest.
