# Supplementary material for: Risk Factors of Venous Thromboembolism in Inflammatory Bowel Disease: A Systematic Review and Meta-Analysis
Source: Front Med (Lausanne). 2021 Jun 28;8:693927. doi: 10.3389/fmed.2021.693927 (PMC8273255; doi:10.3389/fmed.2021.693927)
Supplement: Supplementary file 1 [file Table_1.DOCX]

Supplementary table 1. Sample search strategy

| Database | EMBASE |
| --- | --- |
| Date | 15/01/2021 |
| Strategy | #1 AND #2 AND #3 AND #4 |
| #1 | (‘Bowel Diseases, Inflammatory’ OR ‘IBD’ OR ‘Inflammatory bowel disease’ OR ‘Colitis, Granulomatous’ OR ‘Crohn's Disease’ OR ‘Crohn's Enteritis’ OR ‘Enteritis, Granulomatous’ OR ‘Enteritis, Regional’ OR ‘Ileitis, Regional’ OR ‘Ileitis, Terminal’ OR ‘Ileocolitis’ OR ‘Inflammatory Bowel Disease 1’ OR ‘Regional Enteritis’ OR ‘Colitis Gravis’ OR ‘Idiopathic Proctocolitis’ OR ‘Inflammatory Bowel Disease, Ulcerative Colitis Type’ OR ‘Ulcerative Colitis’)/de OR (Bowel Diseases, Inflammatory OR IBD OR Inflammatory bowel disease OR Colitis, Granulomatous OR Crohns Disease OR Crohns Enteritis OR Enteritis, Granulomatous OR Enteritis, Regional OR Ileitis, Regional OR Ileitis, Terminal OR Ileocolitis OR Inflammatory Bowel Disease 1 OR Regional Enteritis OR Colitis Gravis OR Idiopathic Proctocolitis OR Inflammatory Bowel Disease, Ulcerative Colitis Type OR Ulcerative Colitis); ti,ab |
| #2 | (‘Thromboembolism’ OR ‘Venous thromboembolism’)/de OR (Thromboembolism OR Venous thromboembolism); ti,ab |
| #3 | (‘Case control study’ OR ‘Prospective cohort trial’ OR ‘Retrospective cohort trial’)/de OR (Case control study OR Prospective cohort trial OR Retrospective cohort trial); ti,ab |
| #4 | (‘Risk factors’ OR ‘surgery’ OR ‘Age’ OR ‘gender’ OR ‘steroids’ OR ‘surgery’ OR ‘obesity’ OR ‘BMI’ OR ‘body mass index’)/de OR (Risk factors OR surgery OR Age OR gender OR steroids OR surgery OR obesity OR BMI OR body mass index); ti, ab |
